# A fecal-based test for the detection of advanced adenoma and colorectal cancer: a case-control and screening cohort study

**DOI:** 10.1186/s12916-021-02123-0

**Published:** 2021-10-25

**Authors:** Lian-Jing Cao, Xiao-Lin Peng, Wen-Qiong Xue, Rong Zhang, Jiang-Bo Zhang, Ting Zhou, Zi-Yi Wu, Gai-Rui Li, Tong-Min Wang, Yong-Qiao He, Da-Wei Yang, Ying Liao, Xia-Ting Tong, Fang Wang, Ke-Xin Chen, Shi-Hong Zhang, Li-Qing Zhu, Pei-Rong Ding, Wei-Hua Jia

**Affiliations:** 1 State Key Laboratory of Oncology in South China Guangzhou, Collaborative Innovation Center for Cancer Medicine, Sun Yat-sen University Cancer Center, Guangzhou, People’s Republic of China; 2 Department of Radiation Oncology, Affiliated Hospital of Qingdao University, Qingdao, People’s Republic of China; 3 Shenzhen Nanshan Center for Chronic Disease Control, Shenzhen, People’s Republic of China; 4 Department of Endoscopy and Laser, Sun Yat-Sen University Cancer Center, Guangzhou, People’s Republic of China; 5 Biobank of Sun Yat-sen University Cancer Center, Guangzhou, People’s Republic of China; 6 School of Public Health, Sun Yat-Sen University, Guangzhou, People’s Republic of China; 7 Department of Radiation Oncology, Affiliated Cancer Hospital and Institute of Guangzhou Medical University, Guangzhou, Guangdong People’s Republic of China; 8 Department of Epidemiology and Biostatistics, Key Laboratory of Cancer Prevention and Therapy, Tianjin Key Laboratory of Breast Cancer Prevention and Therapy, Ministry of Education, National Clinical Research Center for Cancer, Tianjin Medical University Cancer Institute and Hospital, Tianjin, People’s Republic of China; 9 Department of Laboratory Medicine, First Affiliated Hospital, Sun Yat-sen University, Guangzhou, People’s Republic of China; 10 Department of Colorectal Surgery, Sun Yat-sen University Cancer Center, Guangzhou, People’s Republic of China

**Keywords:** Colorectal cancer, Advanced adenoma, Noninvasive test, Fecal biomarkers, Screening

## Abstract

**Background:**

Colorectal cancer (CRC) is the leading cause of cancer death worldwide. Screening is a confirmed way to reduce the incidence and mortality rates of CRC. This study aimed to identify a fecal-based, noninvasive, and accurate method for detection of colorectal cancer (CRC) and advanced adenoma (AA).

**Methods:**

Through detection in tissue (*n* = 96) and fecal samples (*n* = 88) and tested in an independent group of fecal samples (*n* = 294), the methylated DNA marker ITGA4 and bacterial markers *Fusobacterium nucleatum* (*Fn*) and *Pepetostreptococcusanaerobius* (*Pa*) were identified from the candidate biomarkers for CRC and AA detection. A prediction score (pd-score) was constructed using the selected markers and fecal immunochemical test (FIT) for distinguishing AA and CRC from healthy subjects by logistic regression method. The diagnostic performance of the pd-score was compared with FIT and validated in the external validation cohort (*n* = 117) and in a large CRC screening cohort.

**Results:**

The pd-score accurately identified AA and CRC from healthy subjects with an area under the curve (AUC) of 0.958, at a specificity of 91.37%; the pd-score showed sensitivities of 95.38% for CRC and 70.83% for AA, respectively. In the external validation cohort, the sensitivities of the pd-score for CRC and AA detection were 94.03% and 80.00%, respectively. When applied in screening, the pd-score identified 100% (11/11) of CRC and 70.83% (17/24) of AA in participants with both colonoscopy results and qualified fecal samples, showing an improvement by 41.19% compared to FIT.

**Conclusions:**

The current study developed a noninvasive and well-validated approach for AA and CRC detection, which could be applied widely as a diagnostic and screening test.

**Supplementary Information:**

The online version contains supplementary material available at 10.1186/s12916-021-02123-0.

## Background

Colorectal cancer (CRC) is the third most commonly diagnosed and the second most fetal cancer, contributing to approximately 10.2% of the annual cancer incidence and 9.2% of the cancer-related mortalities [1, 2]. With the development of socioeconomics and changes in dietary patterns, the incidence and mortality rates of CRC have increased in China in recent years [3].

The underlying neoplastic process from an aberrant crypt to a precursor lesion and eventually to CRC takes 10 to 15 years, providing an optimal window phase for CRC screening [4]. Numerous studies support the fact that screening contributes to the discovery and removal of precursor lesions, represented by adenomas, and early-stage CRC, which could reduce the incidence and mortality rates of CRC. In fact, approximately 63% of CRC deaths could be attributed to the lack of regular screening [5]. Data in the USA show an increase in CRC screening rate from 38% in 2000 to 66% in 2018, which correspondingly resulted in a substantial decrease in CRC incidence and CRC-related mortality [6].

Colonoscopy, a representative structural-based examination, is limited in large-scale population CRC screening due to its high invasiveness and time consumption. FIT is now the most commonly applied noninvasive test due to its easy operation and ability to reduce CRC-related mortality [7]. However, the sensitivity of FIT for early-stage CRC detection, especially for adenomas, is rather unsatisfactory. A meta-analysis demonstrated a sensitivity of 79% (95% CI 0.69 to 0.86) for CRC in average-risk populations [8], while the sensitivity was only 6% to 56% for AA [9]. Thus, in the widely accepted 2-step screening scenario (a simple test finding out those need colonoscopy), the relatively low sensitivity of FIT leads to a relatively high false-negative rate.

The liquid biopsy provides a noninvasive route of sample collection for analysis of tumor-derived DNA, RNA, miRNA, and proteins. A number of studies suggest that analysis of tumor-derived DNA, RNA, miRNA, or proteins can provide relevant information for CRC detection and some tests have been commercially available, including the ColonSentry™ messenger RNA (mRNA) expression panel [10], the Colox® 29-gene panel [11], the CELTiC panel [12], and the *SEPT9* methylated DNA test [13]. However, besides variations regarding the blood collection processing and sample storage, the sensitivity of the abovementioned commercialized kits for identifying CRC from healthy is still have room to improvement.

The stool DNA (sDNA) test, which detects alterations in DNA in tumor cells that slough into the stool, is a new approach for CRC diagnosis and screening. It is a desirable CRC screening method due to its noninvasive, highly sensitive, and user-friendly properties. In 2014, the first sDNA detection kit, Cologuard^TM^ (Exact Science, Madison WI), was approved by the US Food and Drug Administration (FDA) for clinical use [14]. To date, the sDNA test has been recommended as a CRC screening method in the NCCN [15] and Chinese guidelines [16]. For countries with large populations, such as China, it is reasonable to adopt a specific biomarker-based screening kit as a frontline test to select suspected CRC patients and then carry out colonoscopy.

A wide variety of genetic and molecular changes mediate colorectal carcinogenesis, including defective DNA repair, chromosome instability, microsatellite instability, DNA methylation, and microbiota [17]. Many studies have shown compelling evidence that epigenetic markers are ideal diagnostic biomarkers, as they appear quite early in disease pathogenesis [18]. DNA methylation is one of the most ubiquitous epigenetic changes in carcinogenesis [19]. Hypermethylation occurring in CpG islands plays a key pathophysiological role in the initiation and progression of CRC [20]. Scientists have reported many highly sensitive methylated DNA markers for CRC detection and some of them have been commercialized [21–23], including the FDA approved Cologuard^TM^.

Function and application of gut microbiota have been widely explored in recent years. Studies on the pathogenic mechanisms of bacteria have revealed that several pathogenic microbiota contribute to colorectal carcinogenesis. Consistently, the composition of the gut microbiota is different between CRC patients and healthy individuals; some potential protective taxa are decreased, while other procarcinogenic taxa are increased in CRC [24–26]. Although geographically variant, several bacterial species are reproducible and invariably enriched in CRC [27, 28], which pinpoints the potential diagnostic value of detecting a core set of bacteria.

This study aimed to identify a fecal-based, effective, and accurate method for CRC and AA diagnosis and screening. Here, we combined the highly sensitive sDNA test and the highly specific FIT and constructed a prediction model, the pd-score. Moreover, to detect more CRC and AA patients, both CRC-specific methylation markers and pathogenic bacterial markers were included in the sDNA test. The prediction model was well-validated in an external validation cohort and applied in a CRC screening cohort.

## Methods

### Study design

**Fig. 1:**
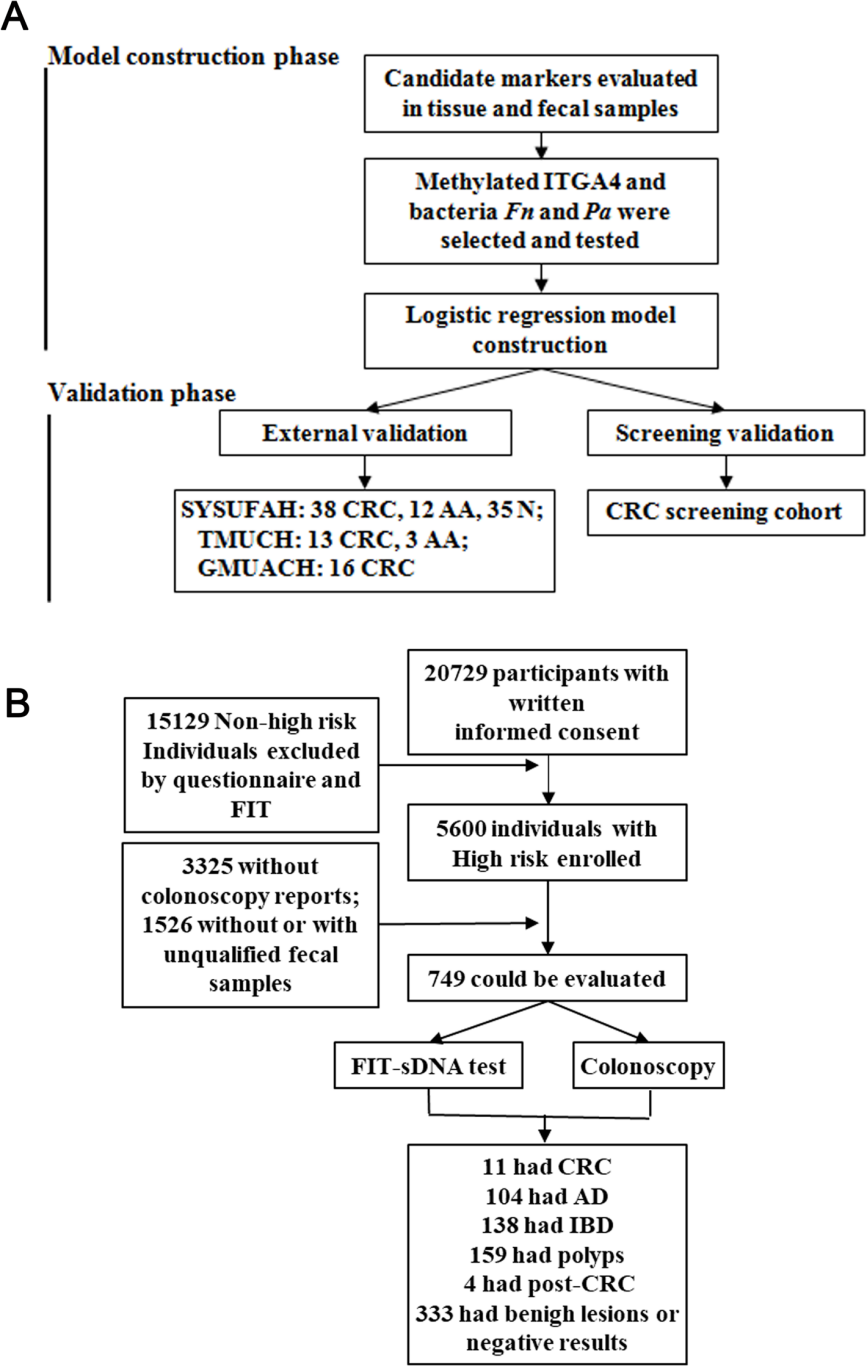
Workflow for model generation and participant enrollment in the CRC screening cohort. **A** Workflow for the selection of methylated DNA, bacterial markers, and prediction model construction. **B** Enrollment and outcomes of the CRC screening cohort

To obtain a reliable CRC diagnosis and screening method, our study consisted of the following two main phases: (1) Model construction phase: the candidate methylated DNA and bacterial markers were first quantified in tissue and fecal samples collected from CRC patients and healthy subjects; the selected markers were then evaluated in an independent group of fecal samples collected from CRC, AA, and healthy subjects; then, the pd-score was constructed for CRC and AA diagnosis using a logistic regression model of the fecal samples mentioned above. (2) Validation phase: The pd-score was validated in the external validation cohort in which fecal samples were collected from three institutions and in a large CRC screening cohort (Fig. 1A, B).

### Study population

Paired cancer and adjacent normal mucosa tissues obtained from 48 stage I–II CRC patients were collected from Sun Yat-sen University Cancer Center (SYSUCC). Fecal samples used in the model construction phase included 195 CRC and 48 AA patients and 139 healthy subjects collected from SYSUCC, which was named the SYSUCC cohort. Fecal samples of the external validation cohort were collected from subjects from three institutions, including 38 CRC and 12 AA patients and 35 healthy subjects from the SYSUFAH, 16 CRC patients from the Affiliated Cancer Hospital and Institute of Guangzhou Medical University (GMUACH), and 13 CRC and 3 AA patients from Tianjin Cancer Institute & Hospital (TMUCH). The demographics of the SYSUCC cohort and the external validation cohort are summarized in Additional file 2: TableS1 and Table S2.

Fecal samples were collected either 1 week before or 1 month after colonoscopy to allow recovery of the gut microbiome [29]. Hospital-based subjects were recruited according to the following criteria: (1) pathologically diagnosed with CRC or AA, (2) the pathological type was adenocarcinoma or adenoma, and (3) no prior history of antitumor treatment. The exclusion criteria were as follows: (1) use of antibiotics within the past 3 months, (2) history of or the presence of any other cancer, (3) a formless fecal sample, (4) inflammatory disease of the gut, and (5) any invasive medical intervention (including resection of adenoma or polyp during colonoscopy, while not only colonoscopy) within the past 3 months. Prior consent from all participants and approval from the Research Ethics Committee of the SYSUCC were obtained for experimentation with human samples.

The stages of the CRC patients were determined according to the 8th edition of the American Joint Committee on Cancer (AJCC) staging system. In our study, the adenoma was diagnosed by experienced pathologist through review of histological features. The adenomas in this study include both advanced adenoma and non-advanced adenoma. Advanced adenomas included the adenomas ≥1 cm along the greatest dimension, or with ≥25% villous histological features, or high-grade dysplasia. Other histologically diagnosed adenomas not meeting these standards were defined as non-advanced adenoma [14]. Proximal CRCs were defined as tumors located in the cecum, ascending colon or hepatic flexure, and distal CRCs were defined as those located in the splenic flexure, descending colon, sigmoid colon, or rectum.

### CRC screening cohort

The CRC screening cohort originated from a subset of the Cancer Screening Program in Urban China [30] and was collected from Nanshan District, Shenzhen city, Guangdong Province, China. Participants enrolled in this study were people aged 45–75 years. All participants were first invited to take a risk assessment using an established Clinical Cancer Risk Score System [30] and FIT. Those who were assessed as positive by the Clinical Cancer Risk Score System or FIT were considered to be at high-risk for CRC and were then recommended to undergo colonoscopy and to provide fecal samples within 90 days after the risk assessment. A total of 20729 participants were enrolled in this study from May 2017 to December 2019. A total of 5600 participants were considered to be at high-risk for CRC, and finally, 749 participants with colonoscopy results and qualified fecal samples could be evaluated. This cohort contained 11 CRC, 104 adenoma (AD), 159 colorectal polyps, 138 inflammatory bowel disease (IBD), 4 postoperative CRC (post-CRC), and 333 other benign lesions or negative results. The demographics of the screening cohort are summarized in Additional file 2: Table S3. This project was approved by the Ethics Committee of the Shenzhen Nanshan Center for Chronic Disease Control, and prior consent was obtained from all participants.

### Fecal sample collection and storage

Fecal samples were collected by two different methods: immediate freezing for hospital-based samples and mixing in fecal preservative buffer (FPB) for the screening samples. Samples from patients and healthy subjects at the hospital were allocated and frozen at −80°C within 4 h after defecation. People enrolled in the screening subject were asked to fill a 2-ml tube containing 1 ml FPB with fecal samples. Buffered samples were delivered to the laboratory and stored at −80°C within 24 h. There was no difference in human genome DNA integrity (Additional file 1: Figure S1A) or bacterial diversity (Additional file 1: Figure S1B-E) between the immediate freezing and buffered samples.

### Marker selection

Seven methylated DNA and five bacterial markers were candidates based on literature review and available CRC detection kit using stool samples. For methylation markers, VIM, BMP3, NDRG4, and SDC2 came from the commercialized CRC detection kits [14, 31–33]. ITGA4 [34], MAL, and CNRIP1 [35] were included because they exhibited good separation of CRC from normal tissues.

For bacterial markers, *Fusobacterium nucleatum* (*Fn*), *Solobacteriummoorei* (*Sm*)*, Pepetostreptococcusanaerobius* (*Pa*), and *Parvimonasmicra* (*Pm*) were included in this study because they were the most reproducible bacterial biomarkers for CRC detection across different datasets [27]. In addition, *Clostridium hathewayi* (*Ch*) was included as it was recently reported to be significantly enriched in patients with colorectal adenoma compared with healthy subjects [36].

### DNA extraction, quantitative real-time PCR of methylated DNA, and bacterial markers

Tissue DNA was isolated with the QIAamp DNA Mini Kit (Qiagen, Valencia, CA) according to the published protocol. Fecal samples (200 mg or 200 μl) were used to isolate human and pathogen DNA with a QIAamp DNA Fecal Mini Kit (Qiagen, Valencia, CA). Briefly, to ensure fecal sample is thoroughly homogenized, we added 5 grinding beads to each stool sample after adding 1 ml InhibitEX buffer, and the samples were vortex continuously for 2 min. And the following operation was according to the manufacturer’s protocol. Tissue- and fecal-derived DNA were chemically modified with sodium bisulfite using an EZ DNA Methylation-Gold kit (Zymo Research, Irvine, CA).

Quantitative real-time PCR amplification was adopted for the measurement of methylation status and the abundances of bacterial markers. For methylation markers, a 20-μl reaction system of EpiTect MethyLight Mix (Qiagen, Valencia, CA) containing 5-μl converted DNA, 250 nM of each primer, and 200 nM of each probe was applied; the thermal cycler parameters of *the LightCycler 480* instrument II were 95°C for 5 min and (95°C for 15 s and 60°C for 1 min) × 45 cycles. Detection of bacterial abundances was performed with LightCycler 480 SYBR Green I Master Mix (Roche, Applied Science) in a 10-μl reaction system containing 30–90 ng DNA and 250 nM of each primer; thermal cycler parameters were 95°C for 5 min and (95°C for 15 s and 60°C for 1 min) × 45 cycles. Nucleotide sequences of the primers and probes are listed in Additional file 2: Table S4 and Table S5 [36–38].

For tissue samples, we use percent of methylated reference (PMR) to assess the methylation level of target genes. Plasmid containing the bisulfate-treated target gene region was constructed and the copy number of each plasmid was calculated according to the following formula: the number of copies of DNA template per μl = (DNA concentration (ng/μl) × Avogadro’s number) / (length of template (bp) × 10e9 × 660). The plasmids were then serially diluted as standards for absolute quantification (10^1^, 10^2^, 10^3^, 10^4^, 10^5^, 10^6^, and 10^7^ copies per μl). Bisulfite-treated CpGenome Universal Methylated DNA was used as positive control. The methylation level of each target gene was calculated as follows: methylation level = (target sample/ ACTB sample) / (target _positive_/ ACTB _positive_) × 100%.

For fecal samples, the quantified CT value was used to directly interpret the level of each methylated gene. Samples without CT values of methylated genes were given a value of 45 to compare the methylation level between different samples. Bacterial markers are presented as abundances and are relative units normalized to 16S rDNA using the 2^−ΔCt^ method (ΔCt = Ct bacterial markers − Ct 16S rDNA and relative abundances = 2^−ΔCt^), which was performed as in a previously described protocol [39]. The relative abundance of each marker is shown as a log value of “×10^8^+1” [36].

### Fecal immunochemical test

The FIT was performed using the Fecal Occult Blood Gold Gel Stripe (W.H.P.M. BIORESEARCH & TECHNOLOGY) according to the manufacturer’s protocol. The minimum allowable detection of hemoglobin was 200 ng per milliliter. Samples were considered positive when color bands appeared simultaneously in the control line and the reaction line; those with only one color band in the control line were determined to be negative, while samples with no color band or only one color band that appeared in the reaction line were regarded as invalid and needed another test. The results are valid within 5 min. The laboratory staff performing the test was experienced and blinded to the colonoscopy results.

### Quality control of the fecal samples

Human HCT116 DKO methylated and nonmethylated DNA (Zymo Research, Irvine CA) were used as positive and negative controls for the PCR system. Samples were considered valid if they satisfied all of the following requirements: (1) CT value of ACTB < 40, (2) CT value of 16S rDNA < 35, and (3) positive control line of the Fecal Occult Blood Gold Gel Stripe in the FIT test.

### Statistical analysis

The Mann-Whitney *U* test was employed to calculate the differences in gene methylation level and bacterial abundance between the two groups of subjects. Kruskal-Wallis H test was performed to estimate the difference of ITGA4 methylation as well as the abundances *Fn* and *Pa* between healthy subjects, advanced adenoma and cancer. The receiver operating characteristic (ROC) curve was used to evaluate the diagnostic performance of markers or models in discriminating patients from healthy subjects. The best cutoff values of the ROC curve were determined by the maximal Youden index (sensitivity+specificity−1). Pairwise comparison of ROC curves was calculated with the method of DeLong et al. [40]. A logistic regression model was used to estimate the performance of marker combinations in discriminating patients from healthy subjects. The pd-score generated from the optimal logistic regression model was calculated as follows: logit (pd-score) = α+β1*ITGA4+β2*Fn+β3*Pa+β4*FIT, where *α* represents the intercept and *β* represents the regression coefficients of each marker. The Mann-Whitney *U* test was used to calculate the differences in the pd-scores of the two groups of subjects. A one-sided McNemar paired-comparisons test was performed to observe the difference in sensitivity between the pd-score and FIT. The chi-square test was applied to assess the association of the detection rate with clinical covariates. Integrated discrimination improvement (IDI) was used to judge the improvement in the pd-score compared with FIT. All hypothesis tests, excluding the McNemar paired-comparisons test, were conducted in a two-sided manner, and a *P* value < 0.05 was considered to be statistically significant. All analyses were performed in R software, version 3.6.3.

## Results

### Identification of the methylated DNA ITGA4 and bacterial *Fn* and *Pa* as diagnostic markers for CRC

**Fig. 2:**
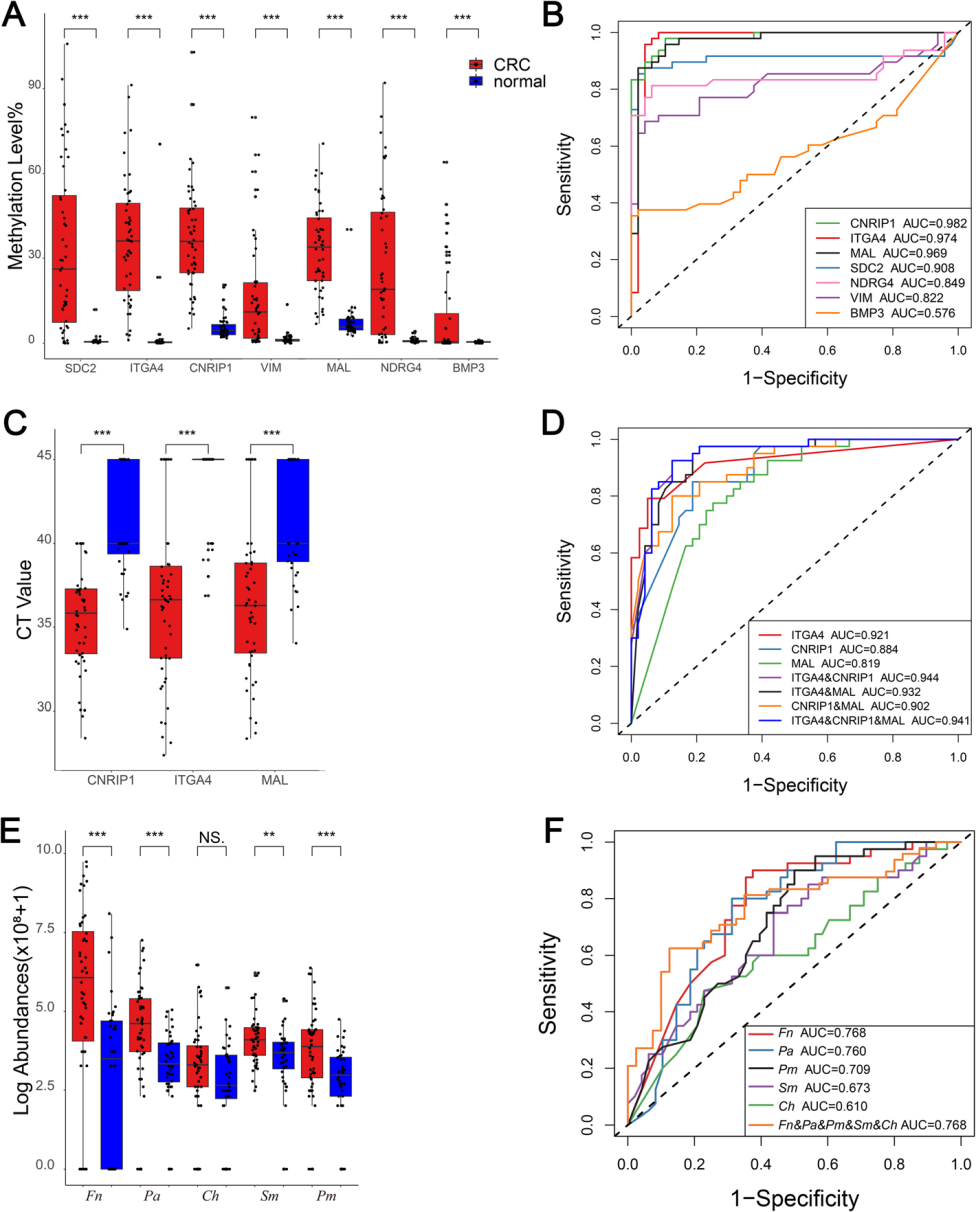
Identification of the methylated DNA ITGA4 and bacterial *Fn* and *Pa* for CRC detection. **A**, **B** Methylation levels (**A**) and ROC curves along with the corresponding AUCs (**B**) of the 7 candidate methylated DNA markers in 48 CRC and adjacent normal tissues. **C**, **D** Methylation levels (**C**) and ROC curves and the corresponding AUCs (**D**) of the 3 selected methylated DNA markers in fecal samples collected from 48 CRC and 40 healthy subjects. **E**, **F** Relative abundance (**E**) and ROC curves along with the corresponding AUCs (**F**) of the 5 candidate bacterial markers in fecal samples collected from 48 CRC and 40 healthy subjects. NS, nonsignificant, ** *P* < 0.01, ***P < 0.001

To identify better methylated DNA markers, we first detected the methylation level of the seven candidate markers in 48 CRC and adjacent normal tissues. The methylation levels of all seven candidates were significantly higher in CRC tissues than in adjacent normal tissues (all *P* < 0.001) (Fig. 2A). Methylated CNRIP1, ITGA4, and MAL showed areas under the ROC curve (AUCs) greater than 0.95 (AUC = 0.982 for CNRIP1, AUC = 0.974 for ITGA4 and AUC = 0.969 for MAL, respectively) and were selected for further testing (Fig. 2B).

To elucidate whether methylation markers that perform well in tissues could be reproduced in fecal samples, we detected methylated CNRIP1, ITGA4, and MAL in fecal samples collected from 48 CRC patients and 40 normal controls. All three markers showed significantly higher aberrant methylation levels in CRC patients than in healthy subjects (all *P* < 0.001) (Fig. 2C). ROC curve analysis demonstrated that ITGA4 had the highest diagnostic efficiency with an AUC of 0.921, but the combination of the three markers (CNRIP1 and ITGA4 and MAL) did not significantly enhance the diagnostic efficiency (AUC = 0.921 for ITGA4 *vs.* AUC = 0.941 for CNRIP1 and ITGA4 and MAL, *P* = 0.163) (Fig. 2D). Meanwhile, 31 out of 40 normal controls showed an absence of ITGA4 methylation, indicating a high specificity of methylated ITGA4 in the fecal samples (Fig. 2C).

We further quantitatively examined the abundances of the five candidate bacterial markers using the fecal samples mentioned above. Among the five candidates, except for *Ch*, the bacterial markers *Fn*, *Pa*, *Sm*, and *Pm* were all significantly enriched in CRC patients compared to healthy subjects (*P* < 0.001 for *Fn*, *Pa* and *Pm*; *P* = 0.005 for *Sm*) (Fig. 2E). ROC curve analysis showed that *Fn* and *Pa* performed better than *Sm* and *Pm* in discriminating CRC from healthy subjects, with AUCs of 0.768 and 0.760, respectively (Fig. 2F). The combination of the five markers (*Fn*, *Pa*, *Ch*, *Pm*, and *Sm*) yielded an AUC value of 0.768, which was not significantly enhanced compared with *Fn* or *Pa* alone.

### Testing of the selected markers in an independent group

**Fig. 3:**
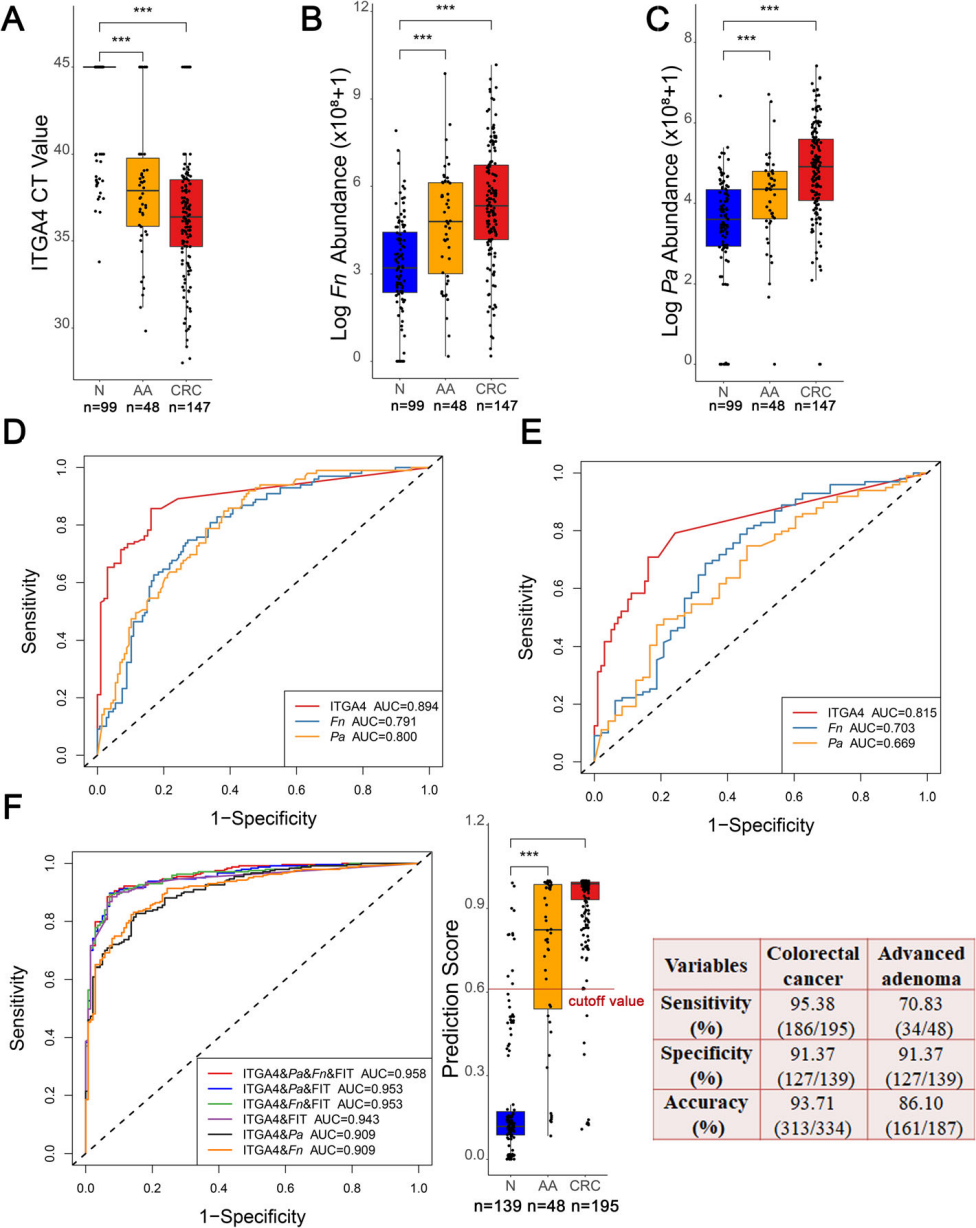
Testing of the selected methylated DNA and bacterial markers and prediction model construction. **A**–**C** Methylation level of ITGA4 (**A**) and relative abundance of *Fn* (**B**) and *Pa* (**C**) in fecal samples collected from healthy subjects (N), AA patients and CRC patients. **D**, **E** ROC curves along with the corresponding AUCs of ITGA4, *Fn*, *Pa*, and FIT in the detection of CRC (**D**) and AA (**E**). **F** ROC curves along with the corresponding AUCs of different combinations of ITGA4, *Fn*, *Pa*, and FIT. **G** Prediction score in normal control and AA and CRC patients generated from the logistic regression model constructed by the combination of ITGA4, *Fn*, *Pa*, and FIT and the diagnostic performance of the pd-score using the cutoff value of 0.6 (determined by the Youden index method)

We further tested the diagnostic reliability of methylated ITGA4 and bacteria *Fn* and *Pa* in an independent group of fecal samples including 147 CRC, 48 AA, and 99 normal controls. Surprisingly, the levels of ITGA4, *Fn* and *Pa* were not only significantly higher in patients with CRC than in healthy subjects (all *P* < 0.001) but also significantly enhanced in patients with AA compared with healthy subjects (all *P* < 0.001) (Fig. 3A–C).

ROC curve analysis demonstrated that ITGA4, *Fn*, and *Pa* exhibited stable diagnostic performance for the detection of CRC, yielding AUCs of 0.894, 0.791, and 0.800, respectively (Fig. 3D). Furthermore, ITGA4, *Fn*, and *Pa* also performed well in discriminating AA patients from healthy subjects, with AUCs of 0.815, 0.703, and 0.669, respectively (Fig. 3E). These results verified the reliability of the diagnostic efficiency of ITGA4, *Fn*, and *Pa.*

### Construction of the prediction model for AA and CRC diagnosis

As combinations of different methylation markers or bacterial markers are unable to significantly improve the diagnostic efficiency, we then considered the possibility that combinations of different types of markers could achieve a better diagnostic performance. A prediction model was constructed using fecal samples from the SYSUCC cohort, including 195 CRC and 48 AA patients and 139 normal controls. The results showed that among all the different combinations, the logistic model constructed with ITGA4, FIT, *Pa*, and *Fn* performed best in discriminating CRC and AA from healthy controls, which was significantly better than any combination of two or three of the four markers. The pd-score obtained according to the four coefficients demonstrated an AUC of 0.958 for CRC and AA detection (Fig. 3F). Using the cutoff of 0.6, as determined by the Youden index method, the pd-score demonstrated a specificity of 91.37% and sensitivities of 95.38% for CRC and 70.83% for AA, respectively (Fig. 3G).

### The pd-score performs significantly better than FIT in diagnosing AA and stage I–II CRC

**Fig. 4:**
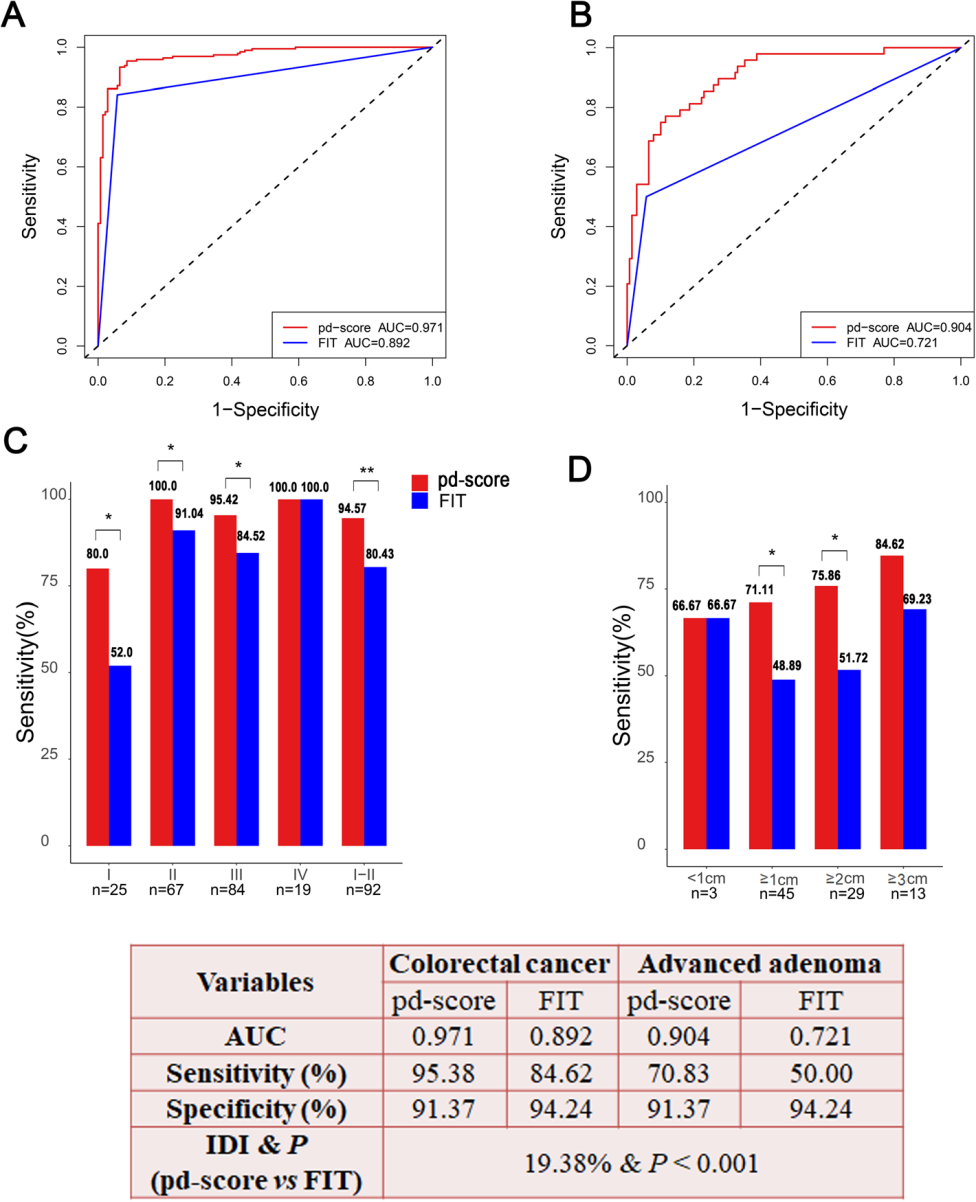
Comparison of the pd-score with FIT in CRC and AA detection. **A**, **B** Comparison of ROC curves along with the corresponding AUCs of the pd-score and FIT in the detection of CRC (**A**) and AA (**B**). **C** Effect of cancer stage on the detection rates of CRC by the pd-score and FIT. **D** Effect of size on the detection rates of AA by the pd-score and FIT. NS, nonsignificant, *P < 0.05, ** *P* < 0.01

FIT has been used for CRC diagnosis and screening for decades, so we assessed the superiority of the pd-score compared to FIT in discriminating CRC and AA from healthy subjects. The pd-score performed significantly better than FIT in discriminating CRC and AA from healthy subjects, with AUCs of 0.971 *vs* 0.892 for CRC and AUCs of 0.904 *vs* 0.721 for AA (Fig. 4A, B). The IDI analysis showed that the diagnostic capacity of the pd-score was 19.38% (95% CI 0.1545–0.2231, *P* < 0.001) higher than that of FIT in discriminating both CRC and AA from healthy subjects. Importantly, the pd-score was significantly more advantageous than FIT in diagnosing stage I–II CRC (sensitivity: 94.57% *vs*. 80.43%, *P* = 0.002) (Fig. 4C) and AA measuring greater than 1 cm (sensitivity: 71.11% *vs*. 48.89%, *P* = 0.016) (Fig. 4D), indicating a possible effective role of the pd-score in CRC screening.

### Validation of the pd-score in the external validation cohort

**Fig. 5:**
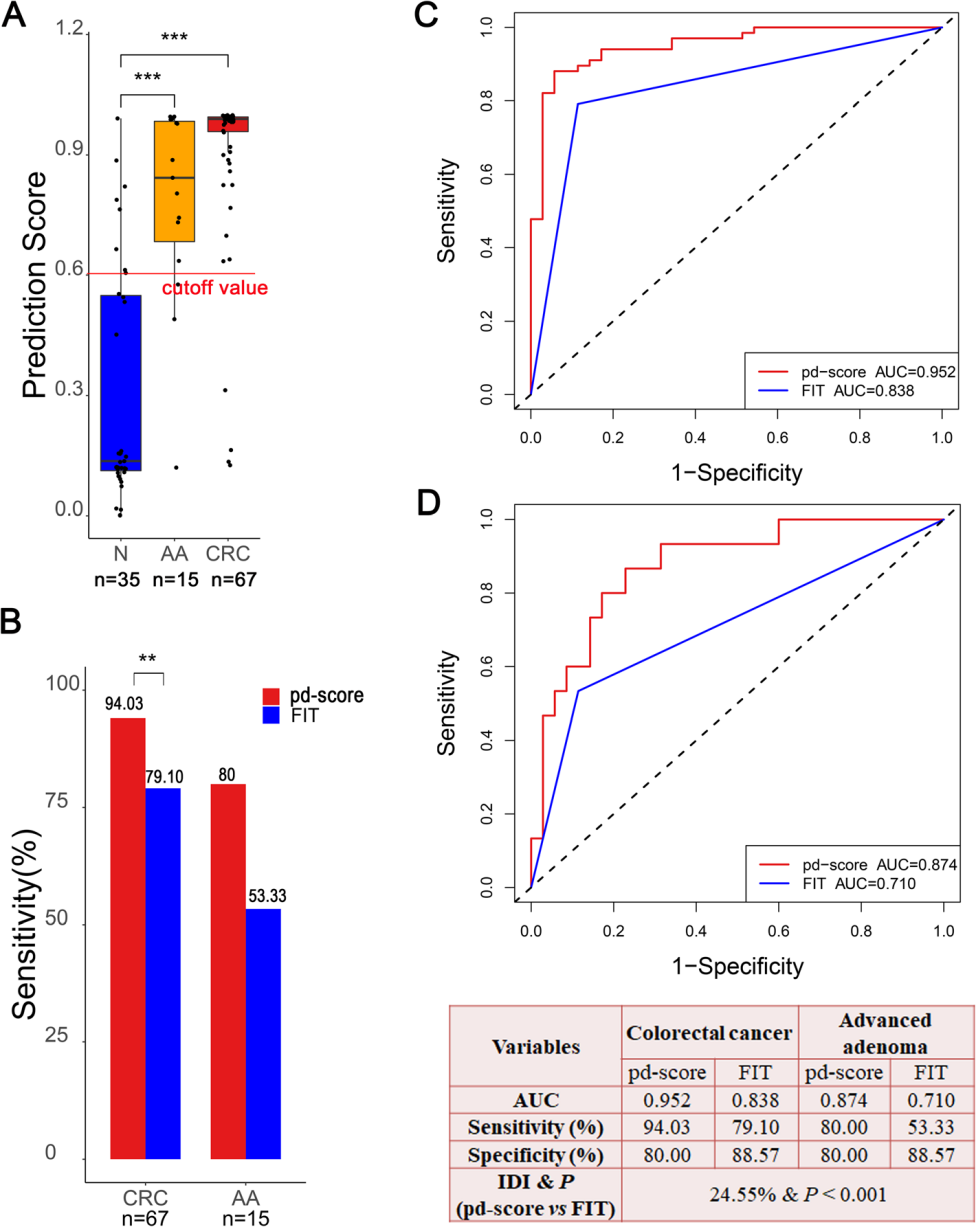
External validation of the pd-score. **A** The prediction score of the normal control and AA and CRC patients generated by the pd-score. **B** Comparison of the detection rates of the pd-score and FIT in CRC and AA. **C**, **D** Comparison of ROC curves along with the corresponding AUCs of the pd-score and FIT in the detection of CRC (**C**) and AA (**D**). ** *P* < 0.01, ***P < 0.001

Consistent with that in the SYSUCC cohort, the pd-scores were significantly higher in AA (*P* < 0.001) and CRC (*P* < 0.001) patients than in normal controls in the external validation cohort (Fig. 5A). Using the same cutoff value generated from the SYSUCC cohort, the pd-score and FIT identified 63 (94.03%) and 53 (79.10%) persons with CRC, 12 (80%) and 8 (53.33%) persons with AA, and 28 (80%) and 31 (88.57%) healthy subjects, respectively (Fig. 5B). The pd-score and FIT demonstrated AUCs of 0.952 and 0.838 for CRC and 0.874 and 0.710 for AA, respectively (Fig. 5C, D). The IDI analysis demonstrated a 24.55% (95% CI 0.1664–0.3254, *P* < 0.001) improvement in the pd-score compared to FIT for discriminating both CRC and AA from healthy subjects.

### Validated screening effect of the pd-score in CRC screening cohort

**Fig. 6:**
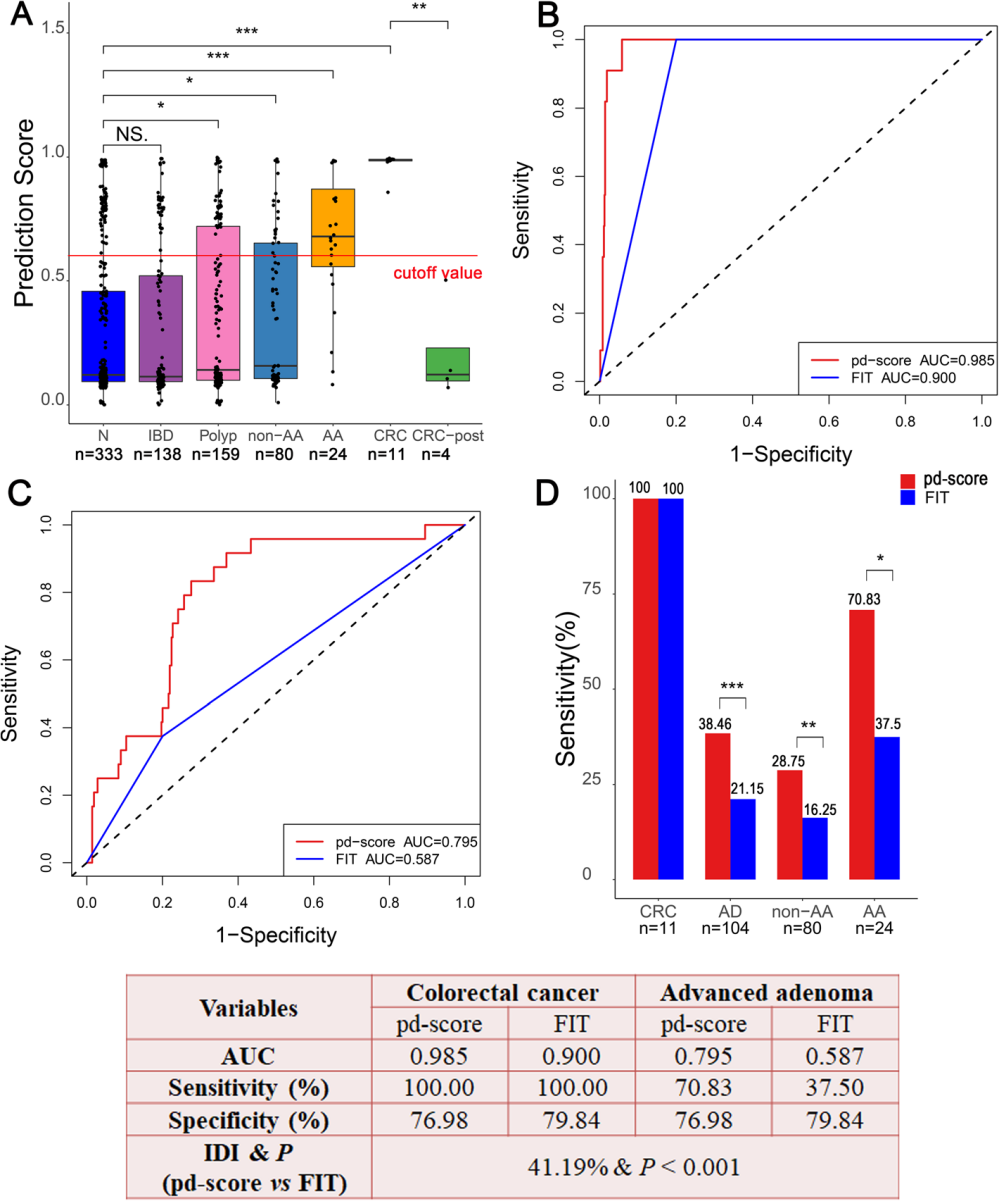
Application of the pd-score in CRC screening in high-risk populations. **A** The prediction score of normal control, IBD, polyp, non-AA, AA, CRC, and CRC-post patients. **B**, **C** Comparison of ROC curves along with the corresponding AUCs of the pd-score and FIT in the detection of CRC (**B**) and AA (**C**). **D** Comparison of the detection rates of the pd-score and FIT for CRC, adenoma (AD), non-AA, and AA. *P < 0.05, ** *P* < 0.01, ***P < 0.001

Among the 749 participants who were eligible for evaluation in the CRC screening cohort, the pd-scores were significantly higher in CRC (*P* < 0.001), AA (*P* < 0.001), nonadvanced adenoma (non-AA) (*P* = 0.012) and polyps (*P* = 0.035) than in healthy subjects. The pd-scores were significantly decreased in postoperative CRC (CRC-post) patients compared with untreated patients (*P* = 0.001) (Fig. 6A). In addition, there was no significant difference in the pd-scores between patients with IBD and healthy subjects (*P* = 0.710). The pd-scores of IBD and polyps were significantly lower than those of AA and CRC (all *P* < 0.001) (Additional file 1: Figure S2), indicating the clinical utility of the pd-score in differential diagnosis.

As the purpose of screening is to identify precancerous lesions and CRC, participants with non-AD and non-CRC were all considered controls in the following analysis. In this context, the pd-score and FIT demonstrated AUCs of 0.985 and 0.900 for CRC (Fig. 6C) and 0.795 and 0.587 for AA (Fig. 6D), respectively. Colonoscopy, the pd-score, and FIT identified 11, 11 (100%), and 11 (100%) persons with CRC; 104, 40 (38.46%), and 22 (21.15%) persons with AD; 80, 23 (28.75%), and 13 (16.25%) persons with non-AA; 24, 17 (70.83%), and 9 (37.5%) persons with AA; and 630, 485 (76.98%), and 503 (79.84%) normal controls, respectively. The pd-score showed a significantly higher sensitivity than FIT in diagnosing AA (*P* = 0.013), non-AA (*P* = 0.004), and AD (*P* < 0. 001) (Fig. 6D). The IDI analysis showed that the pd-score improved the results by 41.19% (95% CI 0.3353–0.4884, *P* < 0.001) compared to FIT in discriminating CRC and AA from normal controls.

## Discussion

The colorectal cancer incidence can be effectively decreased through the discovery and removal of advanced adenomas during screening, and CRC mortality can be remarkably reduced by its detection at an early, resectable stage. This study developed a prediction model using methylated DNA, bacterial markers, and FIT, which showed a high sensitivity for the detection of AA and early-stage CRC.

Strategies for CRC screening have shifted from one step (colonoscopy) to 2 steps, and a 2-step screening scenario is now the most acceptable strategy worldwide [41]. Colonoscopy is the gold standard for the diagnosis of CRC. However, it should be noted that, in addition to its invasive nature and the need for bowel preparation, in countries with large populations, such as China, there are not enough doctors or colonoscopies to meet the needs of one-step screening.

FIT is now the most widely used CRC screening test but it is limited by a relatively low sensitivity for early-stage CRC and AA [42]. In addition, some factors may have impact on the FIT test and lead to false-positive results. A meta-analysis demonstrated that female was related to a significantly higher false-positive rate compared to males. People with medical history of peptic ulcer and anal fissure also tend to have significantly higher risk of false-positive FIT results possibly due to their high risk for bleeding. Using medications of NSAIDs (e.g., aspirin) may leads to higher false positive rate as well [43]. Another population-based screening program conducted in German revealed that older age and new diagnosis of IBD associated with higher risk of false-positive results [44]. Thus, it is rational to combine more specific approaches (sDNA) with FIT to improve the accuracy of CRC screening [14].

It is axiomatic that tests for cancer screening must be sensitive enough, since the primary aim of cancer screening is to filter out precancerous lesions and early-stage cancers [45]. Approximately 85% of CRC cases arise from AA; thus, effectively detecting AA patients is the key in CRC screening. Patients with early-stage CRC have a nearly 90% 5-year survival rate, whereas it is only 14% for those diagnosed with distant disease [6]. Screening out CRC at early stages is crucial for effective surgical and therapeutic interventions. The pd-score showed a sensitivity of 94.57% for stage I–II CRC, which was close to the estimated sensitivity of colonoscopy [46–50]. In the CRC screening cohort, IBD patients were included for analysis and inclusion of these subjects could be closer to the actual scenarios that screening test were applied, since this cohort came from the community. The pd-scores of IBD were significantly different with CRC and were no difference with normal controls. In addition, we computed the specificity for the screening cohort with or without these IBD patients, respectively. The results showed that the specificity is stable (without IBD: 76.83%, with IBD: 76.98%), indicating the combined predictor of pd-score can distinguish CRC from IBD patients.

The high sensitivity of the pd-score is due to the diagnostic complementarity of different types of markers. We performed a subgroup analysis and found high diagnostic complementarity of different markers in different stages and locations of tumors. We found that the detection rates of the four markers (FIT, ITGA4, *Fn*, and *Pa*) all tended to increase with stage (Additional file 1: Figure S3A). Compared with patients at advanced stages, AA and stage I patients are less likely to suffer from tumor hemorrhage; thus, the sensitivity of FIT is limited [14, 51]. In contrast to FIT, methylated ITGA4 is derived from exfoliated cells, and DNA methylation signatures are considered to maintain during colorectal carcinogenesis from adenoma to cancer [52], which may help to explain the significantly higher sensitivity than FIT for AA and stage I patients. Several studies have reported that gut microbial dysbiosis is linked to the progression of colorectal neoplasia [53], and this study observed that the abundances of *Fn* and *Pa* changed with stage. *Fn* showed a sensitivity of 54.17% for AA patients, which contributed greatly to the pd-score of patients with AA. Regarding tumor location, we observed that FIT showed a higher sensitivity for distal CRC, while ITGA4, *Fn*, and *Pa* performed better in discriminating proximal CRC from healthy subjects (Additional file 1: FigureS3B). Hemoglobin from proximal tumors may degrade before reaching the anus, which probably leads to the inferiority of FIT for proximal CRC [54–56]. Differences in developmental origin and environmental mutagens lead to variations in molecular features and gut flora between proximal and distal CRC [57–59]. Previous studies have reported a positive CpG island methylator phenotype (CIMP), and the proportion of *Fn* gradually increased from the rectum to the proximal colon [60–62], which is consistent with our results. Proximal CRC, especially those at an early stage, is difficult to diagnose due to obscure clinical manifestations, and studies have found that the prognosis for patients with proximal CRC is worse than that for patients with distal CRC [63, 64]. Due to the complementarity of markers from different types, the pd-score shows high sensitivity for both AA and CRC, this is of great importance to improve the early diagnosis rate and the survival of proximal CRC patients.

Although the FIT-sDNA test has been accepted and recommended as a CRC screening method by the NCCN [15] and China guidelines [16], the grading of its recommendation is relatively low due to its lower level of supporting evidence. In the Chinese guidelines for screening CRC, the recommendation category of FIT is moderate, but it is low for the FIT-sDNA test [16]. An important reason is that there are few studies revealing the screening effect of the FIT-sDNA test in large populations. Here, in our study, in addition to being validated in multicenter hospital-based samples, our pd-score was also applied in a CRC screening cohort. In multicenter samples, the diagnostic performance of the pd-score improved 24.55% compared with FIT in discriminating CRC and AA from controls. When applied in a screening setting, it achieved a 41.19% improvement in the pd-score compared to FIT in distinguishing CRC and AA from controls. Our study showed that combining FIT and altered fecal DNA yielded higher single-application diagnostic performance than a commercial FIT for both CRC and AA, providing evidence for the application of the FIT-sDNA test in CRC screening and early diagnosis.

This study had obvious strengths. First, this is the first FIT-sDNA test validated in a CRC screening cohort in China. Second, the pd-score constructed in this study showed high sensitivity for advanced adenoma and was well validated in both the multicenter and screening cohorts. Third, the evaluation of the pd-score was performed in comparison to a commercial FIT.

Nevertheless, there are a few limitations to be considered for this study. First, the number of AA samples in the model construction is relatively small, which may lead to a limited power to estimate the sensitivity. However, the sensitivity of the pd-score for AA in the external validation cohort and the CRC screening cohort was more or less in agreement with that in the model construction cohort, indicating the reliability and accuracy of the pd-score in discriminating AA patients from healthy controls. Second, whether the pd-score will maintain good diagnostic performance in other ethnic populations remains unknown. In the end, the biomarkers of our study were selected based on a candidate strategy, thus some effective ones could be missed. Although the validate samples have confirmed its screening efficiency and high potential in practical application, future biomarkers with better performance are still needed to improve the screening efficiency

## Conclusion

The current study provided a noninvasive and convenient approach with high diagnostic accuracy for advanced adenomas and early-stage CRC. Importantly, we constructed a pd-score that was generated from a large case-control cohort and was well validated in an external validation cohort and CRC screening cohort. The capacity for accurate identification of both precancerous lesions and curable-stage CRC will allow the pd-score to be applied widely as a diagnostic method and a screening test.

## Supplementary Information

**Additional file 1: Figure S1.** Effect of the FPB on human genome DNA integrity and bacterial diversity. **Figure S2.** The prediction score of IBD, polyp, non-AA, AA and CRC patients. **Figure S3.** Detection rates of ITGA4, *FN*, *Pa*, FIT and pd-score on different stage and position.

**Additional file 2: Table S1.** Clinical characteristics of the SYSUCC cohort. **Table S2.** Clinical characteristics of the external validation cohort. **Table S3.** Clinical characteristics of the SZ CRC screening cohort. **Table S4.** Primer and probe sequences for quantitative real-time PCR of methylated DNA markers. **Table S5.** Primer sequences for quantitative real-time PCR of bacterial markers.

## Data Availability

The authenticity of this article has been validated by uploading the key raw data onto the Research Data Deposit public platform (www.researchdata.org.cn), with the approval RDD number as RDDA2021001989.

## Abbreviations

AA: Advanced adenoma
AD: Adenoma
AJCC: American Joint Committee on Cancer
AUC: Area under the curve
*Ch*: *Clostridium hathewayi*
CIMP: CpG island methylator phenotype
CRC: Colorectal cancer
FDA: Food and Drug Administration
FIT: Fecal immunochemical test
*Fn*: *Fusobacterium nucleatum*
FPB: Fecal preservative buffer
IBD: Inflammatory bowel disease
IDI: Integrated discrimination improvement
*Pa*: *Pepetostreptococcusanaerobius*
*Pm*: *Parvimonasmicra*
post-CRC: Postoperative CRC
sDNA: Stool DNA
*Sm*: *Solobacteriummoorei*
