# Supplementary material for: A fecal-based test for the detection of advanced adenoma and colorectal cancer: a case-control and screening cohort study
Source: BMC Med. 2021 Oct 25;19:250. doi: 10.1186/s12916-021-02123-0 (PMC8543798; doi:10.1186/s12916-021-02123-0)
Supplement: Supplementary file 1 — Additional file 1: Figure S1. Effect of the FPB on human genome DNA integrity and bacterial diversity. Figure S2. The prediction score of IBD, polyp, non-AA, AA and CRC patients. Figure S3. Detection rates of ITGA4, FN, Pa, FIT and pd-score on different stage and position. [file 12916_2021_2123_MOESM1_ESM.docx]

**Additional file 1**

**A fecal-based test for the detection of advanced adenoma and colorectal cancer: a case-control and screening cohort study**

Lian-Jing Cao ^†1,2^, Xiao-Lin Peng^†3^, Wen-Qiong Xue^†1^, Rong Zhang^†4^, Jiang-Bo Zhang^1^, Ting Zhou^1,5^, Zi-Yi Wu^1^, Gai-Rui Li^3^, Tong-Min Wang^1^, Yong-Qiao He^1^, Da-Wei Yang^6^, Ying Liao^1^, Xia-Ting Tong^6^, Fang Wang^7^, Ke-Xin Chen^8^, Shi-Hong Zhang^9^, Li-Qing Zhu^3^,Pei-Rong Ding^10^, Wei-Hua Jia
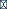
^1,5,6^

^1^Collaborative Innovation Center for Cancer Medicine, State Key Laboratory of Oncology in South China Guangzhou, Sun Yat-sen University Cancer Center, Guangzhou, P. R. China.

^2^Department of Radiation Oncology, Affiliated Hospital of Qingdao University, Qingdao, P. R. China.

^3^Shenzhen Nanshan Center for Chronic Disease Control, Shenzhen, P. R. China

^4^Department of Endoscopy and Laser, Sun Yat-Sen University Cancer Center, Guangzhou, P. R. China

^5^Biobank of Sun Yat-sen University Cancer Center, Guangzhou, P. R. China

^6^School of Public Health, Sun Yat-Sen University, Guangzhou, P. R. China

^7^Department of Radiation Oncology, Affiliated Cancer Hospital and Institute of Guangzhou Medical University, Guangzhou, Guangdong, P. R. China.

^8^Department of Epidemiology and Biostatistics, Key Laboratory of Cancer Prevention and Therapy, Tianjin Key Laboratory of Breast Cancer Prevention and Therapy, Ministry of Education, National Clinical Research Center for Cancer, Tianjin Medical University Cancer Institute and Hospital, Tianjin, P. R. China.

^9^Department of Laboratory Medicine, First Affiliated Hospital, Sun Yat-sen University, Guangzhou, P. R. China.

^10^Department of Colorectal Surgery, Sun Yat-sen University Cancer Center, Guangzhou, P. R. China.

† contributed equally

**Correspondence to:** Wei-Hua Jia, Collaborative Innovation Center for Cancer Medicine, State Key Laboratory of Oncology in South China Guangzhou, Sun Yat-sen University Cancer Center, Guangzhou, P. R. China. Phone: 020-8734-2327; E-mail: [jiawh@sysucc.org.cn](mailto:jiawh@sysucc.org.cn).

**Supplementary Figures S1-S3**

Figure S1: Effect of the FPB on human genome DNA integrity and bacterial diversity.

Figure S2: The prediction score of IBD, polyp, non-AA, AA and CRC patients.

Figure S3: Detection rates of ITGA4, *FN*, *Pa*, FIT and pd-score on different stage and position.

**
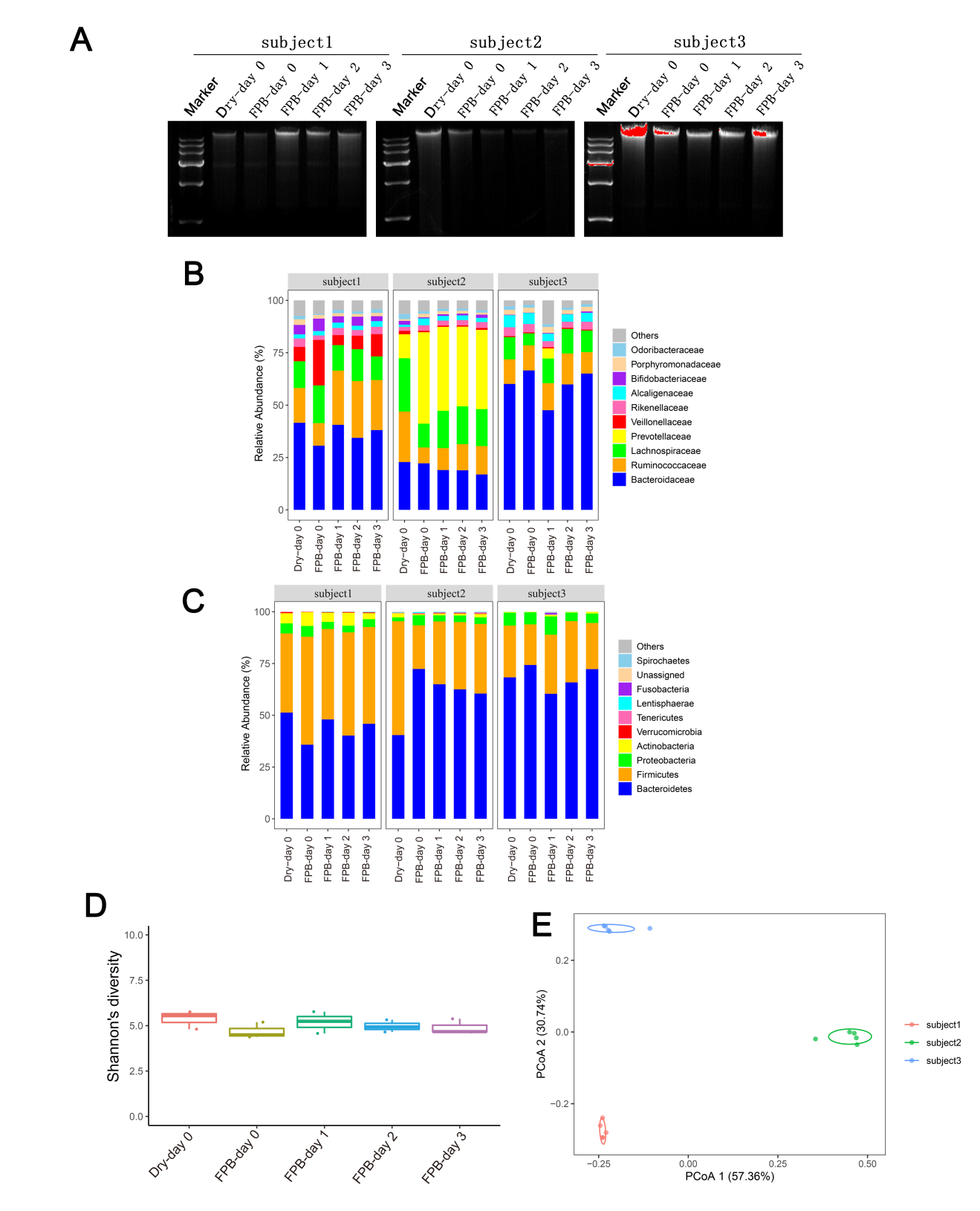
Figure S1.**

**Figure S1. Effect of the FPB on human genome DNA integrity and bacterial diversity. (A)** Effect of the FPB on human genome DNA integrity at different time point in three subjects. **(B, C)** Histogram showing the mean relative abundance at the phylum (B) and the family (C) level. (**D)** Boxplot of Shannon diversity index in each sample group. (**E**) Boxplot of PCoA based on the Bray-curtis distance.

**Figure S2.**


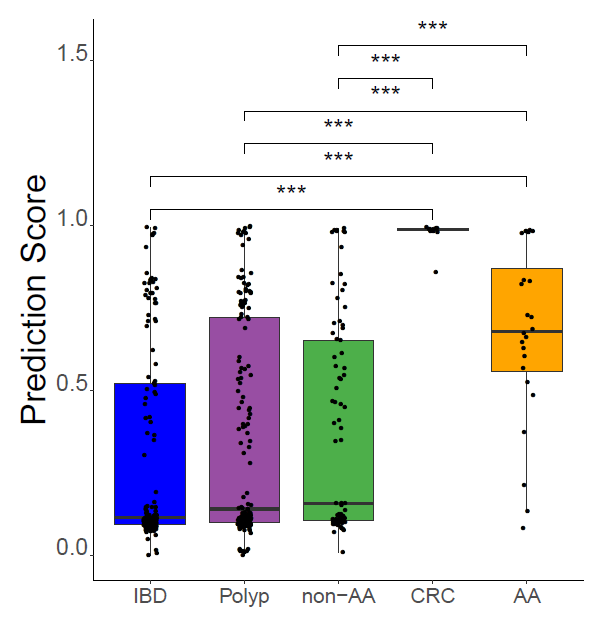


**Figure 2. The prediction score of IBD, polyp, non-AA, AA and CRC patients.**

**Figure S3**


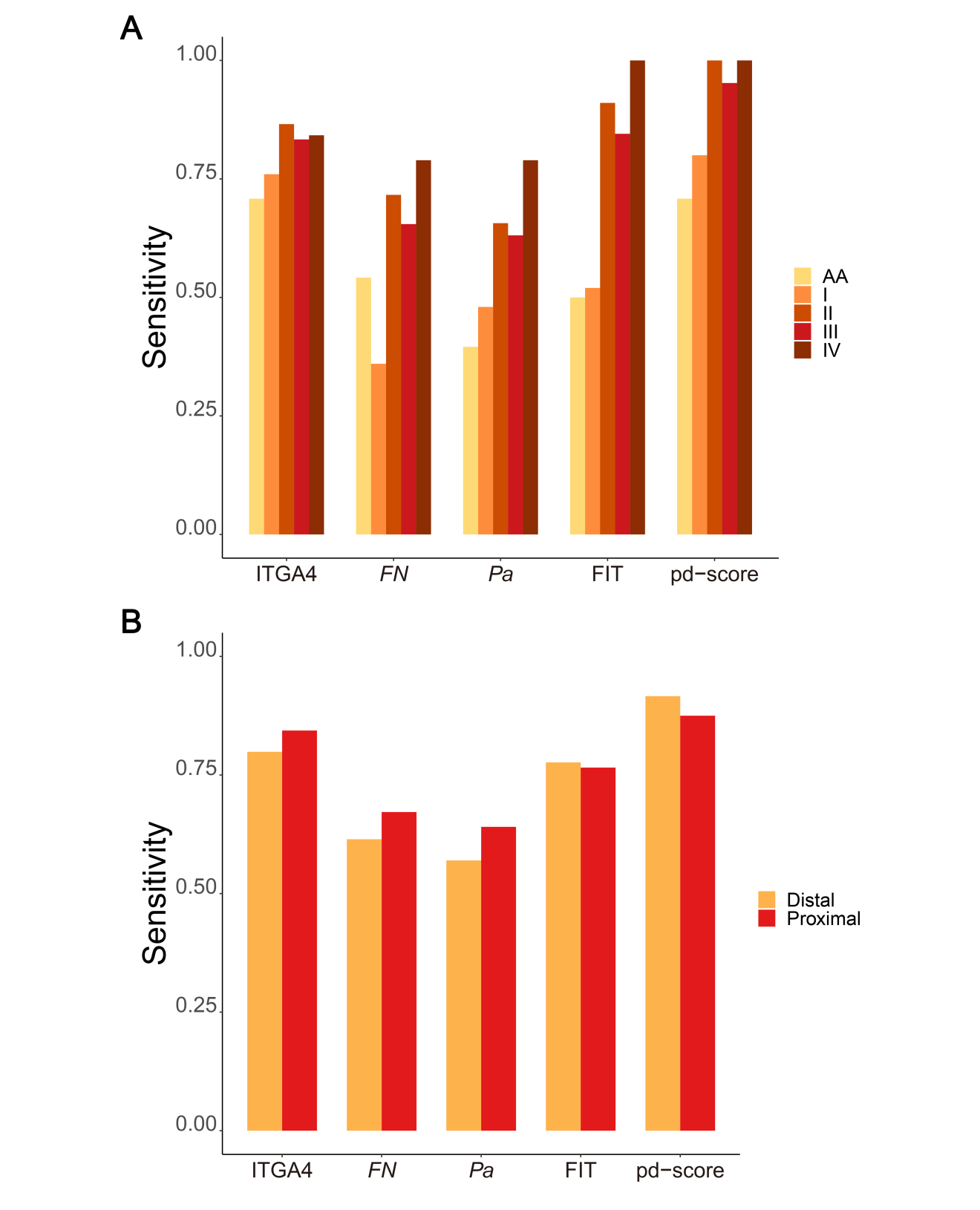
**Figure S3. Detection rates of ITGA4, *FN*, *Pa*, FIT and pd-score on different stage and position. (A)** Detection rates of ITGA4, *FN*, *Pa*, FIT and pd-score on AA and different stages of CRC patients. **(B)** Detection rates of ITGA4, *FN*, *Pa*, FIT and pd-score on distal and proximal CRC patients.
