# Supplementary material for: A fecal-based test for the detection of advanced adenoma and colorectal cancer: a case-control and screening cohort study
Source: BMC Med. 2021 Oct 25;19:250. doi: 10.1186/s12916-021-02123-0 (PMC8543798; doi:10.1186/s12916-021-02123-0)
Supplement: Supplementary file 2 — Additional file 2: Table S1. Clinical characteristics of the SYSUCC cohort. Table S2. Clinical characteristics of the external validation cohort. Table S3. Clinical characteristics of the SZ CRC screening cohort. Table S4. Primer and probe sequences for quantitative real-time PCR of methylated DNA markers. Table S5. Primer sequences for quantitative real-time PCR of bacterial markers. [file 12916_2021_2123_MOESM2_ESM.docx]

**Additional file 2**

**A fecal-based test for the detection of advanced adenoma and colorectal cancer: a case-control and screening cohort study**

Lian-Jing Cao ^†1,2^, Xiao-Lin Peng^†3^, Wen-Qiong Xue^†1^, Rong Zhang^†4^, Jiang-Bo Zhang^1^, Ting Zhou^1,5^, Zi-Yi Wu^1^, Gai-Rui Li^3^, Tong-Min Wang^1^, Yong-Qiao He^1^, Da-Wei Yang^6^, Ying Liao^1^, Xia-Ting Tong^6^, Fang Wang^7^, Ke-Xin Chen^8^, Shi-Hong Zhang^9^, Li-Qing Zhu^3^,Pei-Rong Ding^10^, Wei-Hua Jia
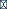
^1,5,6^

^1^Collaborative Innovation Center for Cancer Medicine, State Key Laboratory of Oncology in South China Guangzhou, Sun Yat-sen University Cancer Center, Guangzhou, P. R. China.

^2^Department of Radiation Oncology, Affiliated Hospital of Qingdao University, Qingdao, P. R. China.

^3^Shenzhen Nanshan Center for Chronic Disease Control, Shenzhen, P. R. China

^4^Department of Endoscopy and Laser, Sun Yat-Sen University Cancer Center, Guangzhou, P. R. China

^5^Biobank of Sun Yat-sen University Cancer Center, Guangzhou, P. R. China

^6^School of Public Health, Sun Yat-Sen University, Guangzhou, P. R. China

^7^Department of Radiation Oncology, Affiliated Cancer Hospital and Institute of Guangzhou Medical University, Guangzhou, Guangdong, P. R. China.

^8^Department of Epidemiology and Biostatistics, Key Laboratory of Cancer Prevention and Therapy, Tianjin Key Laboratory of Breast Cancer Prevention and Therapy, Ministry of Education, National Clinical Research Center for Cancer, Tianjin Medical University Cancer Institute and Hospital, Tianjin, P. R. China.

^9^Department of Laboratory Medicine, First Affiliated Hospital, Sun Yat-sen University, Guangzhou, P. R. China.

^10^Department of Colorectal Surgery, Sun Yat-sen University Cancer Center, Guangzhou, P. R. China.

† contributed equally

**Correspondence to:** Wei-Hua Jia, Collaborative Innovation Center for Cancer Medicine, State Key Laboratory of Oncology in South China Guangzhou, Sun Yat-sen University Cancer Center, Guangzhou, P. R. China. Phone: 020-8734-2327; E-mail: [jiawh@sysucc.org.cn](mailto:jiawh@sysucc.org.cn).

**Supplementary Tables S1-S5**

Table S1. Clinical characteristics of the SYSUCC cohort.

Table S2. Clinical characteristics of the external validation cohort.

Table S3. Clinical characteristics of the SZ CRC screening cohort.

Table S4. Primer and probe sequences for quantitative real-time PCR of methylated DNA markers.

Table S5. Primer sequences for quantitative real-time PCR of bacterial markers.

**Table S1**. Clinical characteristics of the SYSUCC cohort.

| **Variables** | **Tissue** | **Fecal** | | | |
| --- | --- | --- | --- | --- | --- |
|  | **CRC** | | **CRC** | **AA** | **Normal** |
| **Gender** | | | | | |
| Male | 25  (52.08%) | 106  (54.36%) | | 26  (54.17%) | 64  (46.04%) |
| Female | 23  (47.92%) | 89  (45.64%) | | 22  (45.83%) | 75  (53.96%) |
| **Age** | | | | | |
| Mean | 59 | 57.29 | | 59.56 | 54.47 |
| Range | 45-74 | 15-89 | | 37-86 | 22-87 |
| **Stage** | | | | | |
| I | 23  (47.92%) | 25  (12.82%) | |  |  |
| II | 25  (52.08%) | 67  (34.36%) | |  |  |
| III |  | 84  (43.08%) | |  |  |
| IV |  | 19  (9.74%) | |  |  |
| **Tumor site** | | | | | |
| Proximal | 11  (22.92%) | 53  (27.18%) | | 16  (33.33%) |  |
| Distal | 37  (77.08%) | 142  (72.82%) | | 32  (66.67%) |  |
| **Adenoma size** | | | | | |
| <1cm |  |  | | 3  (6.25%) |  |
| ≥1cm |  |  | | 45  93.75%) |  |
| ≥2cm |  |  | | 29  (60.42%) |  |
| ≥3cm |  |  | | 13  (27.08%) |  |
| **Total (n)** | 48 | 195 | | 48 | 139 |

CRC: colorectal cancer; AA: advanced adenoma.

**Table S2**. Clinical characteristics of the external validation cohort.

| **Variables** | **SYSUFAH** | | | | **GMUACH** | **TMUCH** | | **Combine** | | |
| --- | --- | --- | --- | --- | --- | --- | --- | --- | --- | --- |
|  | **CRC** | | **AA** | **Normal** | **CRC** | **CRC** | **AA** | **CRC** | **AA** | **Normal** |
| **Gender** | | | | | | | | | | |
| Male | | 19  (50%) | 7  (58.33%) | 19  (54.29%) | 9  (56.25%) | 5  (38.46%) | 0  (0) | 33  (48.25%) | 7  (46.67%) | 24  (68.57%) |
| Female | | 19  (50%) | 5  (41.67%) | 16  (45.71%) | 7  (43.75%) | 8  (61.54%) | 3  (100%) | 34  (50.76%) | 8  (53.33%) | 11  (31.43%) |
| **Age** | | | | | | | | | | |
| Mean | | 60.11 | 69.08 | 52.08 | 57.38 | 57.92 | 62.67 | 59.03 | 67.80 | 54.94 |
| Range | | 24-82 | 55-79 | 15-83 | 30-85 | 44-68 | 46-74 | 24-85 | 46-79 | 24-74 |
| **Total (n)** | | 38 | 12 | 35 | 16 | 13 | 3 | 67 | 15 | 35 |

CRC: colorectal cancer; AA: advanced adenoma.

**Table S3**. Clinical characteristics of the SZ CRC screening cohort.

| **Variables** | **CRC** | **AD** | **AA** | **Non-AA** | **Polyp** | **IBD** | **Normal** |
| --- | --- | --- | --- | --- | --- | --- | --- |
| **Gender** | | | | | |  |  |
| Male | 6  (54.55%) | 46  44.23%) | 13  (54.17%) | 33  (41.25%) | 82 (51.57%) | 47  (34.06%) | 97 (29.13%) |
| Female | 5  (45.45%) | 58  (55.77%) | 11  (45.83%) | 47  (58.75%) | 77  (48.43%) | 91  (65.94%) | 236 (70.87%) |
| **Age** | | | | | |  |  |
| Mean | 59.55 | 60.35 | 60 | 60.45 | 61.83 | 58.70 | 57.50 |
| Range | 45-73 | 45-75 | 46-73 | 45-75 | 45-78 | 45-74 | 40-75 |
| **Total (n)** | 11 | 104 | 24 | 80 | 159 | 138 | 333 |

CRC: colorectal cancer; AD: adenoma; AA: advanced adenoma; non-AA: non-advanced adenoma; IBD: inflammatory bowel disease.

**Table S4**. Primer and probe sequences for quantitative real-time PCR of methylated DNA markers.

| **Target** | **Forward sequence** | **Reverse sequence** | **Probe sequence** |
| --- | --- | --- | --- |
| **VIM** | 5’-AAGTCGCGTTTGGGGGATTTTTAC -3’ | 5’-GACCAAATTATCGCGCTCCACCTC -3’ | FAM-ACCTACCGACGCAACTCCCGCAT -TAMRA |
| **BMP3** | 5’-GTGAGGTTCGCGTAGTTGTTG -3’ | 5’-CGCGACCGAATACAACGAAATAA -3’ | FAM-CCACTTACTACGCTAACCCAACGCA-TAMRA |
| **NDRG4** | 5’-AGGTGTTGGGATATCGGTTG -3’ | 5’-ATTAAAATCCGCACCCCTTC -3’ | FAM-ATTGGGGCGGTTCGGGTTTG- TAMRA |
| **SDC2** | 5’-GTTTATTTTGGGTTTGGTGGTTTGC -3’ | 5’-TCCTCTCGTAACTTCAAACACCCT -3’ | 6FAM-CTCCGCCTAACCCACTCACCGACTC -BHQ1 |
| **ITGA4** | 5’-TATTCGGTCGTGTTGTATAGTTACGG-3’ | 5’-TTCCAACGAACTAACTCTCGCAA -3’ | FAM-CCAACTCTACTCACCATCGATTCGCC-MGB |
| **MAL** | 5’-AAGAGGTTTAGGGCGGTGTT-3’ | 5’-CGCGAAACCCAATAACCTAA-3’ | FAM-TTTTGTTTTTAATTCGCGCG- TAMRA |
| **CNRIP1** | 5’-GTGGAGGGCGTAGTATTGGA -3’ | 5’-AAAACAACGCCGACTACGAC -3’ | FAM-CGCGCGGTTTTGGAGTCGTT- TAMRA |
| **ACTB** | 5’-TTTGGGTTTATTTAGAGTGTAGAT -3’ | 5’-ACCCAACTACCCTAAAACA -3’ | 6FAM-AATCCCTTCCCACCTCCTCAA -TAMRA |

**Table S5**. Primer sequences for quantitative real-time PCR of bacterial markers.

| **Target** | **Forward sequence** | **Reverse sequence** |
| --- | --- | --- |
| ***Fusobacterium nucleatum*** | 5’-CAACCATTACTTTAACTCTACCATGTTCA-3’ | 5’-GTTGACTTTACAGAAGGAGATTATGTAAAAATC-3’ |
| ***Solobacteriummoorei*** | 5’-CTCAACCCAATCCAGCCACT -3’ | 5’-TATTGGCTCCCCACGGTTTC -3’ |
| ***Pepetostreptococcusanaerobius*** | 5’-AGACGAATTCAAGTCAGTAAATACA-3’ | 5’-CTCCTATCCACCAGGATATCAA-3’ |
| ***Parvimonasmicra*** | 5’-GTCACTACGGAAGAATTTGTC-3’ | 5’-GGCTTGAGCGATAATAACTTC-3’ |
| ***Clostridium hathewayi*** | 5’-GGGCTGCGGAAGCAACTTA-3’ | 5’-GATGACCTCGCCCTGATCAT-3’ |
| **16s rDNA** | 5’-GCAGGCCTAACACATGCAAGTC-3’ | 5’-CTGCTGCCTCCCGTAGGAGT-3’ |
